# Highly Directional Sonar Beam of Narwhals (*Monodon monoceros*) Measured with a Vertical 16 Hydrophone Array

**DOI:** 10.1371/journal.pone.0162069

**Published:** 2016-11-09

**Authors:** Jens C. Koblitz, Peter Stilz, Marianne H. Rasmussen, Kristin L. Laidre

**Affiliations:** 1 Bioacoustics Network, Neuss, Germany; 2 Bioacoustics Network, Hechingen, Germany; 3 The University of Iceland’s research center in Húsavík, Húsavík, Iceland; 4 Polar Science Center, Applied Physics Laboratory, University of Washington, Seattle, United States of America; Institute of Deep-sea Science and Engineering, Chinese Academy of Sciences, CHINA

## Abstract

Recordings of narwhal (*Monodon monoceros*) echolocation signals were made using a linear 16 hydrophone array in the pack ice of Baffin Bay, West Greenland in 2013 at eleven sites. An average -3 dB beam width of 5.0° makes the narwhal click the most directional biosonar signal reported for any species to date. The beam shows a dorsal-ventral asymmetry with a narrower beam above the beam axis. This may be an evolutionary advantage for toothed whales to reduce echoes from the water surface or sea ice surface. Source level measurements show narwhal click intensities of up to 222 dB pp re 1 μPa, with a mean apparent source level of 215 dB pp re 1 μPa. During ascents and descents the narwhals perform scanning in the vertical plane with their sonar beam. This study provides valuable information for reference sonar parameters of narwhals and for the use of acoustic monitoring in the Arctic.

## Introduction

Cetacean species in the Arctic ecosystem are subject to broad-scale ecological changes as a result of sea ice loss and climate warming [1]. Arctic sea ice has been decreasing in extent and thickness since 1990 [2]. Model simulations indicate a continuing retreat and the possibility of ice-free summers in the Arctic Ocean within a few decades [3]. These climate related changes will result in large increases in natural resource development, marine shipping, transportation and infrastructure. They will be accompanied by longer seasons of navigation for tankers, passenger ships, fishing vessels, and government and commercial icebreakers [2] and an increased presence of the marine tourism industry. Furthermore, a growing worldwide demand for natural resources has the Arctic poised as a significant contributor to the global economy as a provider of hydrocarbons, hard minerals and fisheries.

The additional burdens from increased anthropogenic activities are very likely to amplify the already negative impacts from Arctic ecosystem change. In particular increases in anthropogenic sound sources will likely interfere with important biological functions for cetaceans such as foraging, migration, communication, and predation avoidance [4]. Therefore, it is a critical time to obtain a good understanding of the baseline ecological and behavioral relationships between Arctic cetaceans, their environment and how they use sound.

Narwhals are one of only two species of toothed whales that inhabit waters above the Arctic Circle (67°N) year round. Narwhals are an important representative species for understanding increasing noise in the Arctic with loss of sea ice and how these items will have potential behavioral and ecological effects. Most of the world’s narwhals inhabit the Baffin Bay/Davis Strait pack ice in winter. They make extensive annual migrations from high Arctic summering grounds in West Greenland and high Arctic Canada to offshore wintering grounds, where >80,000 narwhals (over 80% of the world’s population) occupy dense pack ice between November and April [5–8]. Narwhals make minimal horizontal movements on these wintering grounds and feed intensively on the bottom where a major portion of the annual energy intake is obtained [9–11].

Narwhals inhabit a complex environment where hearing and processing sounds serve critical biological functions related to communication, foraging, reproduction, navigation and predator-avoidance [4]. Our study focused on narwhals in the pack ice of Baffin Bay where they overwinter. We collected some of the first recordings of this species in this habitat using a 16-channel vertical array deployed from leads in the pack ice. We used the data to characterize and quantify sonar parameters such as ASLs, directionality, changes in emission direction, and the spectral composition of the echolocation beam. This research can be a model for developing baselines of Arctic cetacean behavioral ecology in the context of predicted increases in human activities and anthropogenic sound.

## Materials and Methods

**Fig 1:**
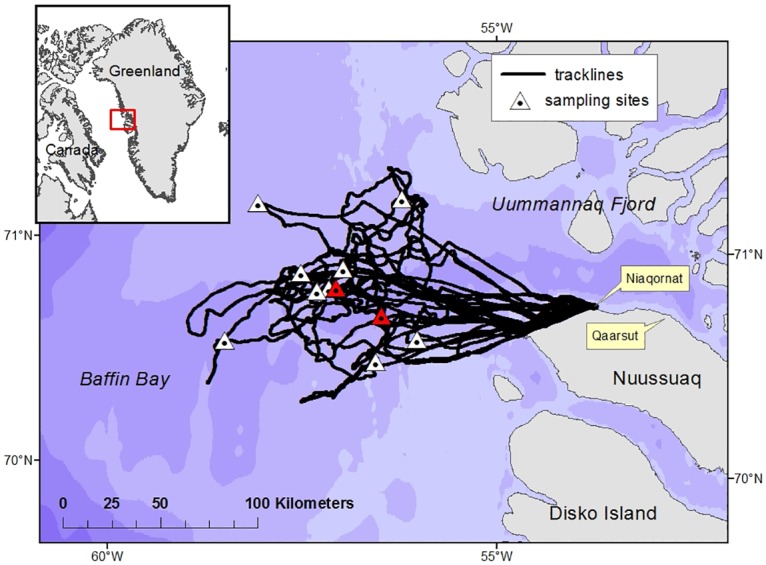
Map of location of sampling sites and search effort tracklines flown in West Greenland in spring 2013. Triangles show all sites sampled in this study; red triangles show sites 10 and 11.

Aerial searches for narwhals were carried out from an AS350 helicopter (Air Greenland) on seven days between March 21^st^ and March 31^st^ 2013 based out of Niaqornat, West Greenland. On clear weather days the helicopter was flown 100 to 150 km offshore in the pack ice using strategically placed fuel depots while observers searched for narwhals in the vicinity of the leads and cracks in the sea ice (Fig 1). When whales were spotted, the helicopter landed on the sea ice close to the lead and the array was deployed from the edge of the lead for periods of 10 minutes to several hours, depending on conditions. Narwhals were visually observed during all recordings within a maximum distance of 1 km from the array. Average air temperatures were -20°C and sea ice was >98% concentration.

### Recording set-up

An array of 16 Reson TC-4013-5 hydrophones (sensitivity -215 dB re 1V/μPa; flat (+/-2 dB) frequency response between 1 and 150 kHz) was used to record narwhal echolocation signals. The 16 receivers were oriented vertically in a linear vertical array, attached to a line (diameter 2 mm) and spaced 1 m apart. The topmost receiver was 3 m below the water surface, the lowest receiver at 18 m depth. The hydrophone array was kept vertical by 4 kg of weight attached to the end of the line.

The hydrophone signals were amplified by 38 dB using a custom made 16 channel amplifier, no high pass filter was used and the hydrophones served as a low pass filter (150 kHz, 1 pole). Each hydrophone was calibrated, and the resulting frequency response of each channel known. Simultaneous analog to digital conversion was performed by National Instrument A/D converter with 16 bit resolution at a sampling rate of 500 kHz per channel (National Instruments PXI-6123). The clipping level of the entire recording chain was 206 dB pp re 1 μPa at 100 kHz and the frequency response between 1 and 150 was flat (+/-2 dB).

Multiple channels were visualized during the recordings by the custom made software MALTA (Microphone Array Localization Tool for Animals, CAE Software & Systems). On six days the array was deployed at eleven independent locations to record narwhals and recordings were made continuously for up to 2:31 h per location. In total, 10:32 hours (550 GB of data) were recorded. Recordings were chunked (loss-less) to 5 s long wav-files for optimal post-processing. The narwhal data reported in this study were selected from two locations (sites 13_10 and 13_11), for a total of 1:39 h of recordings. We only report data from these two sites in this study for all parameters for sake of consistency. Recordings made at a third site (site 13_16) were used to measure the localization accuracy.

### Data Analysis

#### Localization

**Fig 2:**
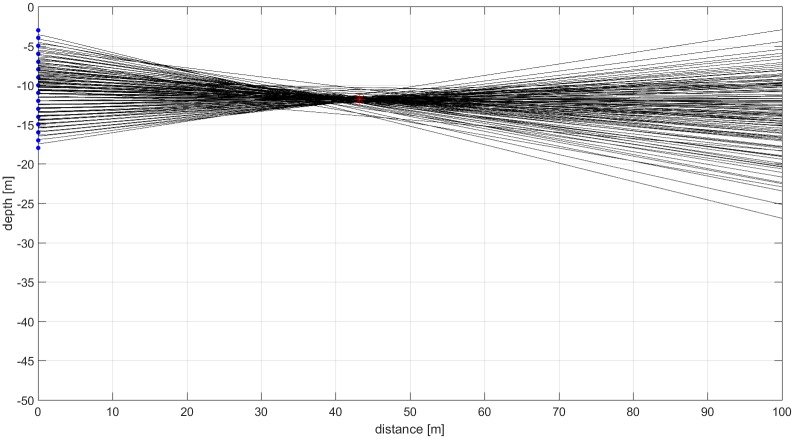
The receiver positions of the vertical hydrophone array are indicated by blue dots. The red star indicates the position based on the least square method. Each hyperbole is based on the time delay of a single hydrophone pair.

Each recording was screened visually for the presence of narwhal clicks. Only recordings with clicks present were chosen for subsequent analysis. For each click recorded with a received level (RL) of 146 dB pp re 1 μPa, i.e. with sufficient signal to noise ratio (SNR >12 dB), the position of the source at click emission was computed by measuring the time of arrival difference (TOAD). First the cross correlations of all 120 possible receiver pairs were performed. The receiver resulting in the best cross correlation with the 15 other receivers was chosen as a template for subsequent processing. The TOADs between the template channel and each of the remaining 15 channels was computed using the cross correlations of short signal sections containing the clicks. Based on these 15 TOADs, the overdetermined source position was calculated based on a least square error optimization [12]. In order to exclude erroneous localizations due to artifacts, up to three channels whose TOADs represented the largest and unreasonable discrepancies in relation to their calculated source distance were excluded from source localizations in a recursive process. For most clicks, the source position was calculated based on all 16 channels; however in case of disturbances at least 13 channels were used. The source position obtained by the least square method and a maximum of 120 hyperbole based on the TOADs were plotted to verify each localization (Fig 2). Data with good SNR from two recording locations were excluded since the source position computed based on the least square method did not coincide with the intersections of the hyperbole. This is presumably due to the bending of the array due to strong winds and currents.

#### Localization accuracy and error assessment

Echolocation signals were played back using a transducer at depths of 5 and 13 m and distances between 13 and 150 m to the array at location 13_16. The true distance between the transducer and the array was measured with a laser range finder. The depth of the source was localized with high precision at all distances. The localized distance between array and transducer was between 80 and 90% of the true distance between 13 and 50 m and at 60 to 70% at ranges of 100 and 150 m. The environmental conditions at the site where localization accuracy was determined were representative for all sites where recordings were made. Acoustic localization resulted in an underestimation of the true distance to the source at the larger distances investigated here. This is in accordance with other studies where a linear hydrophone array has been used to measure the distance to a sound source [13–17]. Assuming this error is indeed a one sided error, the underestimation of the true distance to the source results in an underestimation of the ASL. The measured ASL, given the error measured here, is between 1 to 5 dB lower than the true ASL. The measured beam width is also influenced by the localization error as the beam width estimation is in good approximation inverse proportional to the distance estimation. When underestimating the distance to the source, the beam width is overestimated. Based on the localization error combined with the distances we recorded animals at and the estimated beam width, the true beam width is likely 1–2° narrower than reported here, hence we are reporting conservative measures of the directionality.

The hydrophones used in this study are omnidirectional in the horizontal plane but not in the vertical plane. Given that animals were recorded at unknown bearings to the array, arranging them vertically reduces the influence of vertical directionality.

#### Assigning clicks to echolocation sequences

Due to the decreasing localization accuracy at larger distances, only clicks localized up to a distance of 100 m were considered. All localizations and metadata were visualized in a Matlab routine and each click positioned was screened for localization accuracy. Clicks with little error according to within least square localization and good matches between the least square method and intersection of hyperbole were manually assigned to an echolocation sequence based on the temporal patterning of the click emission, the RL and the localized distance and depth. Each echolocation sequence originates from one individual only but multiple sequences from one individual can be recorded.

#### Measurements of sonar parameters

**Fig 3:**
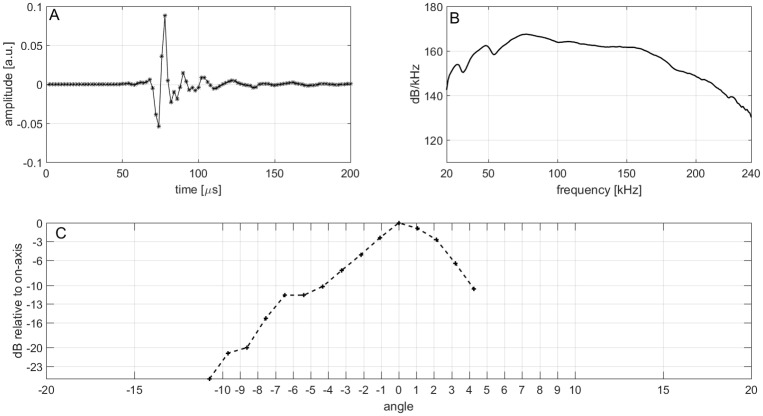
Narwhal echolocation click recorded at second 62 with the maximal amplitude on hydrophone 12 (see Fig 6). Waveform (A) showing sampling points, spectra (B) and vertical beam pattern (C). Negative angles show pattern above the beam axis, positive angles the pattern below the beam axis. The ASL is 215 dB pp re 1 μPa, the -3 dB BW is 3.5° (1.3° above and 2.2° below the beam axis).

For the subsequent analysis, signals were bandpass filtered (4 pole, high pass at 20 kHz, low pass at 240 kHz). The receiver’s individual sensitivity was considered when performing the analysis of sonar parameters. We report the apparent source level according to Møhl et al. [18], the ASL is stated as the peak-peak (pp) measure of the clicks Hilbert transformation relative to 1 μPa at 1 meter source distance. As the signals are very short and broadband, sampling at 500 kHz possesses the risk of not measuring the peak amplitude correctly (Fig 3), however using the Hilbert transformation of the signals can approximately reconstruct this. The ASL was computed using the sonar equation based on the derived animal’s distance to each of the receivers, a transmission loss of 20 log(r) and an absorption of 0.03 dB/m [19].

A click was only considered for further analysis if the maximal intensity was not recorded at one of the outermost receivers. Any click reported on-axis vertically could however be a tangential section of the beam in the horizontal plane. By selecting the click with the highest intensity from every track, while assuming that the animal performs scanning in the horizontal plane and keeps the source level constant, one on-axis click from every track was selected for further analysis. The intensity distribution was then interpolated using all 16 receivers and the direction of maximal intensity was termed on-axis. All subsequent off-axis measurements were made relative to this direction. In order to be considered for final analysis a click had to fulfill the following criteria following Villadsgaard et al. [20] and Ladegaard et al. [21]: (i) it had to be part of a click train of at least nine clicks, (ii) the RL had to exceeded 146 dB pp re 1 μPa, (iii) the clicks were localized at distances closer than 100 m with good localization accuracy as indicated by intersecting hyperbolas (iv) the maximal intensity of the click was not directed towards the upper or lower end of the array and (v) only the most intense click from each track was chosen for final analysis.

### Ethical statement

This study was conducted in accordance with IACUC procedures as approved by the University of Washington (#4155–01, PI Laidre) and the US Office of Naval Research. Permission to conduct research in Greenland waters was provided to K. Laidre by the Government of Greenland and Greenland Institute of Natural Resources, Nuuk.

## Results

**Fig 4:**
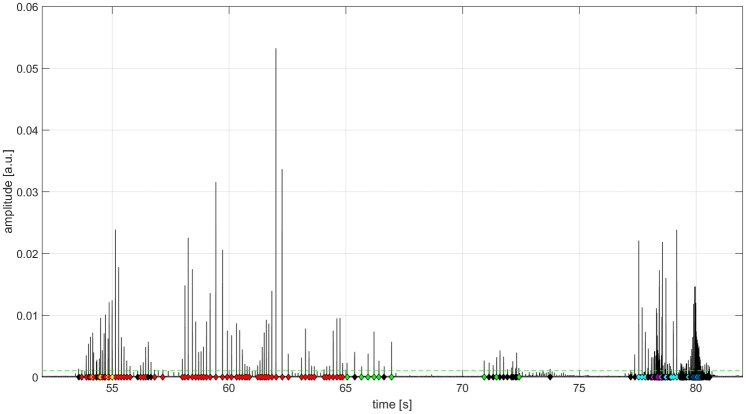
Hilbert transform of the received amplitude of a 30 second long recording at one of the central array hydrophones. The different colored dots indicate the 6 different sequences that a total of 106 clicks were assigned to that were positioned less than 150 m away. Overlapping click trains could be separated based on the special patterning of the localizations. Dashed green line shows detection threshold of 146 dB pp re 1 μPa.

**Fig 5:**
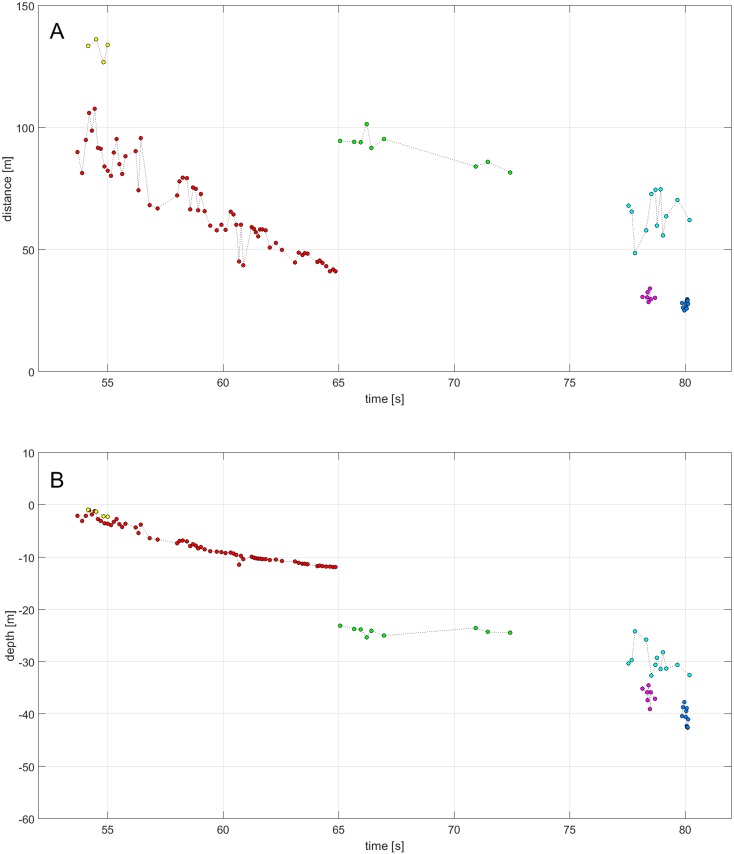
Localized distances to the array (A) and depth (B) of clicks assigned to 6 tracks originating from 2–6 animals. Data of 63 clicks assigned to track one (shown by red dots) will be used in further figures.

Narwhals were visually present in the area when all recordings were made, and no other species occupies the offshore Baffin Bay pack ice at that time of year. Thus, all clicks were assumed to be recorded from narwhals. At both sampling sites the localizations and the ICI clearly indicated that multiple individuals were recorded at once, regular clicks of highly varying received levels were recorded. In a 30 second long recording made on March 27^th^ 2013 and used as an example in the subsequent analysis, clicks were assigned to six different tracks of varying length originating from two to six individual narwhals (Figs 4 and 5).

In 1:39 hours of recordings on two days, 3492 clicks above a threshold of 146 dB pp re 1 μPa were localized. Most of these clicks were directed towards the lower end of the array or below, making beam and source level measures impossible. A total of 94 clicks emitted in 11 tracks were suitable for detailed analysis, one click per track was chosen for final beamwidth and ASL analysis.

### Dive behavior

The positions of the clicks resulted in dive tracks of individual narwhals. All animals were tracked close to the surface at depths of less than 70 m and only localizations closer than 100 m were considered. Recordings were obtained during ascents and descents while animals were approaching the array or moving at equal distance to it (Fig 5).

### Scanning behavior

**Fig 6:**
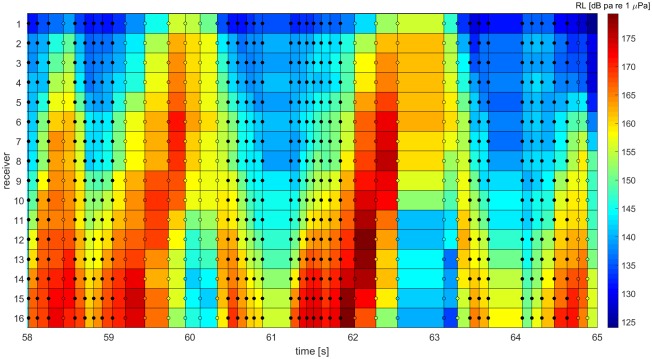
Distribution of the received level (indicated by different colors) of 42 clicks emitted by a single animal at the 16 array hydrophones. The animal does not direct its beam at a single receiver but performs vertical scans over the array. The width of each section indicates the inter-click-interval. Filled circles indicate a click that is directed towards the edge of the array. Those clicks were not considered for further analysis.

The direction of signal emission varied between clicks, indicating that the animal scanned the water column. The emission direction was indicated by the maximal received intensity. Often clicks were directed towards the lower end of the array but scanning movements from the lowest receiver to the topmost receiver was observed (Fig 6).

### Beam width and ASL

**Fig 7:**
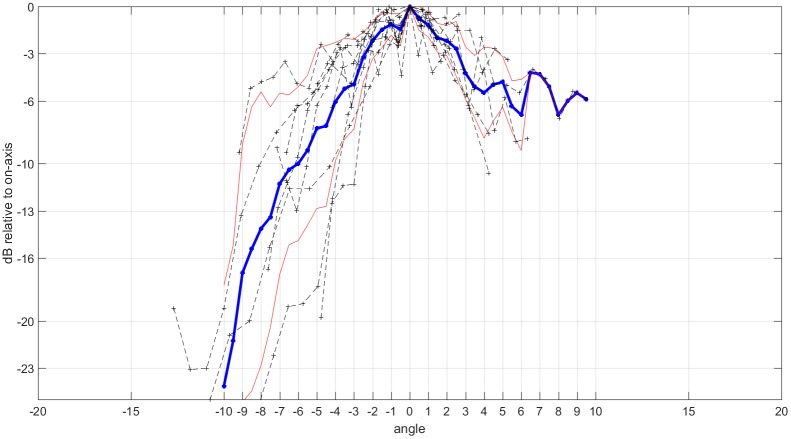
Single click beam patterns of 11 on-axis clicks from 11 tracks (black). Average beam pattern (blue) and standard deviation (red). Negative angles show pattern above the beam axis, positive angles the pattern below the beam axis. The -3 dB BW is 5.0° (2.4° above and 2.6° below the beam axis).

The -3 dB beam width (- 3 dB BW) of a single click is 3.5° (1.3° above and 2.2° below the beam axis, Fig 3). The composite -3 dB BW of 11 on-axis clicks is 2.4° above the beam axis and 2.6° below the beam axis, resulting in an overall -3 dB BW of 5.0° (Fig 7). Source level measurements show high click intensities of up to 222 dB pp re 1 μPa. The average ASL of 11 clicks is 215 dB (std: 6) No correlation between ALS or beam width to the distance to the array nor depth were present.

### Waveform and spectra

**Fig 8:**
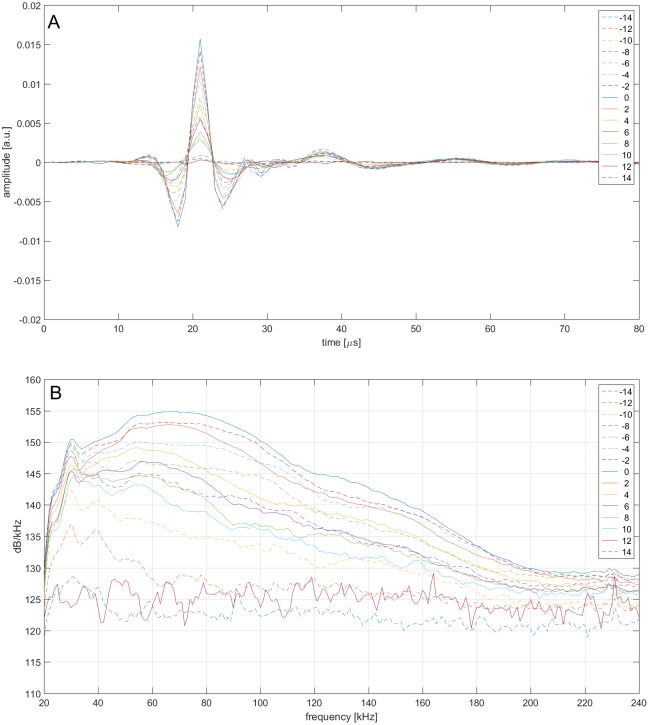
Averaged click waveforms (A) and spectra (B) for 94 clicks received at the 16 hydrophones at various angles between +15° and -15° relative to on-axis.

**Fig 9:**
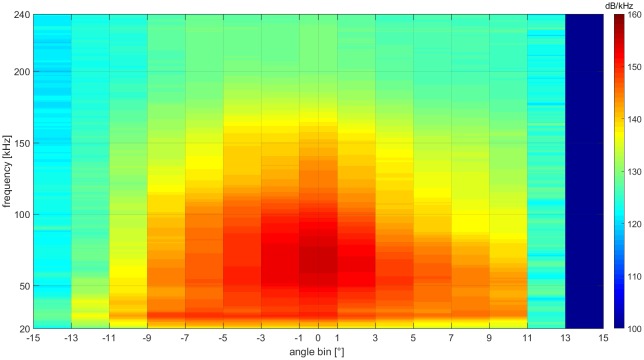
Vertical angular dependent spectral variation relative to on-axis based on 94 clicks. Highest intensities and highest frequencies are emitted in the on-axis direction. High frequency content and intensities decay when recording off the acoustic axis. Negative angles: above the beam axis, positive angles: below the beam axis.

For each click on-axis in the vertical plane, the waveforms and spectra were averaged in 2° wide bins relative to the on-axis direction of the click, showing the spectral variation at various angles relative to on-axis (Figs 8 and 9).

Based on 11 clicks recorded on-axis in both planes, the average peak frequency is 71 kHz, the -3 dB bandwidth 31 kHz and the duration 18.3 μs (Table 1).

**Table 1:** Sonar parameters of 11 on-axis narwhal clicks.

	Mean ± s.d.
Duration_-10 dB_ [μs]	18.3 ± 3.7
ASL [dB pp re 1 μPa]	215 ± 6
F_p_ [kHz]	71.3 ± 15.1
-3 dB bandwidth [kHz]	31.1 ± 8.7
-10 dB bandwidth [kHz]	81.5 ± 25.4
-3 dB beamwidth [°]	5.0
Number of clicks	11

## Discussion

Based on a recent broad-ecological review, the narwhal was identified as one of the most sensitive Arctic marine mammals [22] given its specialization, limited geographic range and narrow habitat niche. Thus, in order to better predict the impacts of increasing anthropogenic activities and climate change, baseline data are needed which provide information on narwhal movements, habitat selection, foraging and acoustic ecology. The narwhal emits clicks with high directionality to achieve high intensities in the forward direction and possibly to reduce clutter echoes. This narrow sonar beam is used to scan the environment with successive clicks allows this species to thrive in the complex acoustic environment of the Arctic. The data presented here might allow differentiating between clicks from the two arctic toothed whale species, the beluga and the narwhal. This is a first crucial step towards acoustic monitoring in this inaccessible environment.

### Narwhal sounds

The narwhal is one of the first toothed whales for which click source parameters were measured in the field using a hydrophone array [23]. Since then, most likely to the logistical challenges associated with field recordings in the high Arctic, comparatively few studies have been carried out. Most studies that have been conducted were limited a priori by the recording setup to lower frequencies. Clicks and whistles up to 24 kHz were first described [24, 25], with Watkins et al. [24] acknowledging that they did not cover the full repertoire of narwhal. More recent studies using single receivers resulted in the description of whistles, pulsed calls, and clicks [26, 27]. Recordings with higher sampling rates revealed the true broadband nature of narwhal clicks where train clicks contained energy at above 100 kHz [28] and even extending above 200 kHz [29]. Using a vertical hydrophone array, Møhl et al. [23] measured source levels of broadband narwhal clicks of up to 227 dB pp re 1 μPa (sensu [30]. An increasing use of multi hydrophone arrays allowed for more detailed and additional measures of the biosonar properties from free-ranging toothed whales such as the apparent source levels (ASLs) and the variation thereof [14, 31]; the directionality of the emitted clicks [13, 32, 33] and variation thereof [34, 35]; and the click spectra on-axis and at various angles off-axis [32, 36]. Recent publications on other species report higher directionalities, ASLs and frequency contents [32, 37] than have been reported before because earlier studies were conducted on captive animals with different characteristics [20, 32]. In addition to an increase in studies on animals in the field, larger and more sophisticated recording systems have been used in the past decade.

### Data quality

By using stringent criteria the sample size was reduced drastically to 11 clicks considered for the final analysis. Only clicks localized at a distance of 100 m or closer and with a RL of 146 dB pp were considered for click parameter analysis. Clicks directed towards the edge of the array (usually the lowest most hydrophone) were then excluded from subsequent analysis and only the most intense click from each track was considered for final analysis.

The underestimation of the distance in the calibration trials and the not considered erroneous click localizations at some recording locations was likely caused by the array bending under the surface of the pack ice in strong water currents. The localization errors in the analyzed dataset do influence the DI measurements. Since the distance to the source during the calibration trials was always underestimated, the measured narwhal ASLs and directionalities should also be considered underestimates.

Since the animals were scanning over the array in the vertical domain, the maximal intensity was directed at different receivers, making it unlikely that a single miscalibrated receiver caused to influence the beam width measurements.

We assumed that the recorded animals were swimming dorsal up during the first part of the ascent and descent [38], and therefore we assume to have measured above the beam axis when measuring above the maximal intensity and below the beam axis when measuring below the maximal intensity.

### High directionality

The measured average -3 dB BW of 5.0°, corresponding to a DI of 31 dB [derived using the approximate relationship, -3 dB BW = 185° x10^(-DI/20)^ [39, 40]] makes the narwhal click the most directional biosonar signal reported to date. The most directional echolocation signals of smaller toothed whales have been reported for the other species endemic to the Arctic, the beluga [41] having a -3 dB BW of 6.5°. Most other smaller toothed whales emit sonar beams with a -3 dB BW between 8 and 13° [16, 30, 32, 33, 37, 42–44], whereas the largest toothed whale, the sperm whale (*Physeter macrocephalus*) emits signals with a -3 dB BW of 8.3°[45] Recent studies using larger hydrophone arrays and at least in some studies free ranging animals instead of stationary animals have in the harbour porpoise (*Phocoena phocoena*) resulted in higher directionalities as described earlier [33, 34, 37]. Smaller toothed whales all show similar directionalities which is driven by two factors: the size of the emitter and the emitted frequency. Larger emitter and higher frequencies are capable of producing higher directionalities. Small odontocetes can achieve high directionality by emitting a signal at high frequencies, large ones produce signals with the same directionality emitting clicks with lower frequencies. The directional beam is advantageous for a deep diver like the narwhal with the possibility to detect prey items at long distances while reducing clutter from the pack ice or water surface.

### Asymmetric beam

The echolocation beam is not symmetric in the vertical plane. Both, the analysis of the composite beam and single click beam measurements indicate a wider beam shape below the beam axis compared to the beam shape above the beam axis. Most studies on the directionality of toothed whale or bat echolocation signals assume a symmetrical echolocation beam. Few studies have investigated horizontal asymmetry and discussed it in respect to the mechanisms of click production [37, 46, 47]. Dorso-ventral beam asymmetry has been observed in the harbor porpoise [37], the false killer whale (*Pseudorca crassidens*) [44], bottlenose dolphins (*Tursiops* spp.) [48–50] and the beluga [41]. In all species, except the beluga, the beam was narrower above the beam axis than the below the beam axis. It is thus unlikely that the asymmetry of the narwhal’s beam is caused by the tusk but rather a consequence of the functional anatomy of a toothed whales head. It may be speculated that it is an evolutionary advantage for toothed whales to have a narrower beam above the beam axis. A narrower beam above the beam axis would reduce echoes from the water surface or pack ice. In addition, the emission direction relative to the longitudinal axis of Blainville’s beaked whales (*Mesoplodon densirostris*) [51] and the false killer whale [44] are directed downwards, further reducing echoes from the surface.

### Vertical scanning behavior and source levels

Bats and toothed whales use highly directional signals to increase the detection distance in the forward direction and reduce clutter from the periphery. To sufficiently sample the volume ahead of them, the moving animals direct their clicks or calls in different directions for subsequent signals. This scanning behavior has been studied in captive bats [52, 53], bats in the field [54] and toothed whales in captivity [55–57], Only limited data is available on the scanning behavior of whales in the field [51]. The scanning behavior allows to increase the sampling volume while searching for prey but also to direct the narrow sonar beam relative to single prey items to optimize localization in the final stage of the approach [53]. The scanning in the vertical domain of narwhals over a large hydrophone array shows the resulting changes of changing the emission direction of the echolocation signals. The observed scanning could be caused by head movements as seen in dolphins [57] and beaked whales [51], by beam steering where the emission direction changes without coupled head movements [58], or by a combination of both. Using the highly directional beam to scan the sea ice from below might be an optimal strategy to help localize open water in ~98% sea ice coverage. The maximal source levels reported here are 6 dB less intense than the first maximal ASLs measurements by Mohl et al. [23]. The narwhal emits clicks with intensities comparable to the ones by delphinids [59–61].

### Angular dependent spectral variation

Narwhals emit very broadband clicks containing considerable energy at frequencies above 200 kHz. These high frequencies have only recently been reported for narwhals [29] scanning over a single receiver. In part this is due to the use of recording systems with low sampling rate unsuited to record these high frequencies in previous studies. However the high directionality in combination with the shown scanning makes it inherently difficult to record clicks on the acoustic axis. The intensity at lower frequencies is emitted less directional as has been shown for delphinids [32, 36, 43], however no clear patterns of spectral notches as have been described for bottlenose dolphins [32, 36] were present in the narwhal clicks. The angular dependent spectral variation is likely to be driven by the click spectra and structures in the animals head such as air sacs, skull and melon [62, 63]. This might be species-specific and aid in species identification of recorded clicks beyond the parameters used previously such as temporal and spectral information [64–67].

### Future effects of anthropogenic change

Baffin Bay, West Greenland is an abyss up to 2,300 m and essentially an “acoustic bowl” where narwhals have developed highly specialized acoustic sensory systems to find their prey at depths of >1500 m. Narwhals have very high site fidelity to winter feeding areas in this area and over 80% of the world’s narwhals spend the winter in this area. Their summering refuge for some of these whales (i.e., Lancaster Sound) is the site of a future year-round shipping route that is expected to be operational as a consequence of sea ice loss (Northwest Passage). Information reported in this study can inform future work on the use of sound by narwhals and the potential impacts of increasing anthropogenic noise in the Arctic. Finley et al. [68] reported on visual reactions of belugas and narwhals to ice-breaking ships in the Canadian High Arctic and noted narwhals demonstrated a “freeze response”. This suggests narwhals’ site fidelity and lack of behavioral plasticity may not allow them to evacuate areas where anthropogenic noise sources will mask communication and acoustic orientation. Future work elucidating details on the use of sound by narwhals will assist in predicting the potential impacts of Arctic change [69].
